# PlasmidTron: assembling the cause of phenotypes and genotypes from NGS data

**DOI:** 10.1099/mgen.0.000164

**Published:** 2018-03-12

**Authors:** Andrew J. Page, Alexander Wailan, Yan Shao, Kim Judge, Gordon Dougan, Elizabeth J. Klemm, Nicholas R. Thomson, Jacqueline A. Keane

**Affiliations:** ^1^​ Infection Genomics, Wellcome Sanger Institute, Wellcome Genome Campus, Hinxton, Cambridge, UK; ^2^​ Department of Medicine, University of Cambridge, Cambridge, UK; ^3^​ London School of Hygiene and Tropical Medicine, London, UK; ^†^​ Present address: Quadram Institute Bioscience, Norwich Research Park, Norwich, UK.

**Keywords:** plasmids, *de novo* assembly, genome-wide association study, mobile genetic elements, antimicrobial resistance

## Abstract

Increasingly rich metadata are now being linked to samples that have been whole-genome sequenced. However, much of this information is ignored. This is because linking this metadata to genes, or regions of the genome, usually relies on knowing the gene sequence(s) responsible for the particular trait being measured and looking for its presence or absence in that genome. Examples of this would be the spread of antimicrobial resistance genes carried on mobile genetic elements (MGEs). However, although it is possible to routinely identify the resistance gene, identifying the unknown MGE upon which it is carried can be much more difficult if the starting point is short-read whole-genome sequence data. The reason for this is that MGEs are often full of repeats and so assemble poorly, leading to fragmented consensus sequences. Since mobile DNA, which can carry many clinically and ecologically important genes, has a different evolutionary history from the host, its distribution across the host population will, by definition, be independent of the host phylogeny. It is possible to use this phenomenon in a genome-wide association study to identify both the genes associated with the specific trait and also the DNA linked to that gene, for example the flanking sequence of the plasmid vector on which it is encoded, which follows the same patterns of distribution as the marker gene/sequence itself. We present PlasmidTron, which utilizes the phenotypic data normally available in bacterial population studies, such as antibiograms, virulence factors, or geographical information, to identify traits that are likely to be present on DNA that can randomly reassort across defined bacterial populations. It is also possible to use this methodology to associate unknown genes/sequences (e.g. plasmid backbones) with a specific molecular signature or marker (e.g. resistance gene presence or absence) using PlasmidTron. PlasmidTron uses a *k*-mer-based approach to identify reads associated with a phylogenetically unlinked phenotype. These reads are then assembled *de novo* to produce contigs in a fast and scalable-to-large manner. PlasmidTron is written in Python 3 and is available under the open source licence GNU GPL3 from https://github.com/sanger-pathogens/plasmidtron.

## Data Summary

1. Source code for PlasmidTron is available from GitHub under the open source licence GNU GPL 3; (url – https://github.com/sanger-pathogens/plasmidtron).

2. Simulated raw reads files have been deposited in figshare; (url – https://doi.org/10.6084/m9.figshare.5406355.v1).

3. *Salmonella enterica* serovar Weltevreden strain VNS10259 is available from GenBank; accession number GCA_001409135.

4. *Salmonella enterica* serovar Typhi strain 60006 is available from GenBank; accession number GCA_900185485.

5. Accession numbers for all of the Illumina datasets used in this paper are listed in the supplementary tables.

**Impact Statement**

PlasmidTron utilizes the phenotypic data normally available in bacterial population studies, such as antibiograms, virulence factors, or geographical information, to identify sequences that are linked to the phenotype.

## Introduction

When defining bacterial populations through whole-genome sequencing (WGS), the samples often have detailed associated metadata that relate to disease severity, antimicrobial resistance, or even rare biochemical traits. When comparing these bacterial populations, it is apparent that some of these phenotypes do not follow the phylogeny of the host, i.e. they are genetically unlinked to the evolutionary history of the host bacterium. One explanation for this phenomenon is that these signals relate to genes that are moving independently between hosts and therefore are likely associated with mobile genetic elements. However, identifying the element that is associated with these traits can be complex, especially if the starting point is short-read WGS data from which they can be difficult to assemble due to repeats. This means that, despite the increased use of next-generation WGS in routine diagnostics, surveillance and epidemiology, this type of association is relatively unexplored.

Recently a number of methods have been developed to address the problem of assembling mobile genetic elements from next generation sequencing (NGS) data [1]. plasmidSPAdes[2] detects plasmids by analysing the coverage of assembled contigs to separate out chromosomes from plasmid-like sequences. By filtering the dataset, a higher qualityassembly is possible. However, if the copy number of the plasmids is similar to that of the chromosome, it is difficult to separate out candidate plasmid contigs. Unicycler [3] is a hybrid assembler that can combine short- and long-read data to produce fully circularized chromosomes and plasmids. It essentially fixes many of the deficiencies of SPAdes[4] and fine-tunes it for assembling bacteria. Recycler [5] takes an assembly graph and aligned reads to search for cycles in the graph that may correspond to plasmids. The method is only partially implemented, with substantial work required on the researcher's part to generate input files in the correct formats. It is shown to work well on small simple plasmids; however, it does not scale to larger more complex plasmids. All of these software applications utilize SPAdes within their methods, work on a single sample at a time, and require no *a priori* knowledge about the samples themselves. placnet can additionally be used for plasmid reconstruction from WGS data [6]; however, as noted in [7], the workflow includes a manual pruning step, so it was excluded from the present evaluation of fully automated tools.

**Fig. 1.**
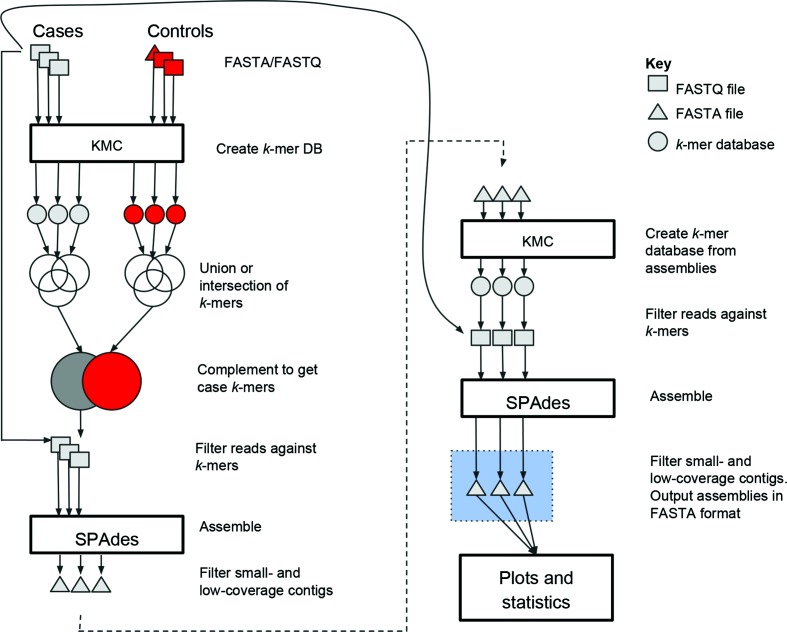
The PlasmidTron algorithm. FASTQ files are denoted as squares, FASTA files as triangles and *k*-mer databases as circles.

We present PlasmidTron (Fig. 1), which utilizes the phenotypic data normally available in bacterial population studies, such as antibiograms, virulence factors, or geographical information, to identify sequences that are mobile and where their distribution is independent of the host phylogeny and matches the phenotypic metadata recorded for a given sample set. Given a set of reads, categorized into cases (showing the phenotype) and controls (phylogenetically related but phenotypically negative), PlasmidTron can be used to assemble *de novo* reads from each sample linked by a phenotype. A *k*-mer-based analysis is performed to identify reads associated with a phenotype. These reads are then assembled *de novo* to produce contigs. By utilizing *k*-mers and only assembling a fraction of the raw reads, the method is fast and scalable to large datasets, whilst also producing more accurate assemblies. This approach has been tested on plasmids because of their contribution to important pathogen-associated traits, such as antimicrobial resistance (AMR) (hence the name), but there is no reason why it cannot be utilized for any sequence that can move independently through a bacterial population. The method is tested on simulated and real datasets drawn from *Salmonella enterica* and *Klebsiella pneumoniae*,and compared with other methods, and the results are validated with long-read sequencing. PlasmidTron is a command-line tool, is written in Python 3 and is available under the open source licence GNU GPL3 from https://github.com/sanger-pathogens/plasmidtron.

## Methods

PlasmidTron (Fig. 1) takes two spreadsheets as input, one containing paired-end read file names in FASTQ format for samples displaying the phenotype (trait), the other containing FASTA or FASTQ file names for samples not displaying the phenotype (non-trait). The input files can be optionally ‘gzipped’ to minimize disk usage. For each sample in both the traits and non-traits, all *k*-mers are calculated using kmc (syntax versions v2.3.0 or v3.0.0) [8, 9] and the frequency of occurrence of each *k*-mer is counted. The *k*-mer size defaults to 51 and can be optionally set to any odd number in the range 21–127 (assembler limitation). This produces a database of *k*-mer counts for each sample, which will be used in the next step. In the case of paired-end reads, both the forward and reverse are included in the same database for a sample. If raw reads in FASTQ format are provided as input for a sample, *k*-mers occurring fewer than five times in a sample are filtered out, since *de novo* assembly is more error-prone below this level of *k*-mer coverage. If sequences in FASTA format are provided as input for a sample, no filtering is applied.

All of the *k*-mer count databases for the trait samples are combined using standard set operations provided bykmc[8, 9]. The default set operation is ‘union’ where *k*-mers observed in any sample are combined into a single *k*-mer count database. The stricter ‘intersection’ set operation can optionally be used, which creates a single *k*-mer count database where a *k*-mer must be present in every trait sample. Similarly, all of the *k*-mer count databases for the non-trait samples are combined with the ‘union’ set operation to create a single *k*-mer count database of non-traits. Any *k*-mer occurring in the non-trait database is removed from the trait database, leaving a database of *k*-mers that are unique to the traits only. In the case of repetitive elements, such as transposons, if they exist in the non-traits database, they will be excluded from the traits database; however, if they are less than the insert size they may be re-included in the final scaffolding step.

The raw reads in FASTQ format, plus their mates, which match any of these unique trait *k*-mers, are extracted from each trait sample using FASTAQ (v3.15.0) (https://github.com/sanger-pathogens/fastaq). Optionally the user can define a percentage *k*-mer coverage (default 20 %) that a read must have to be considered, and also if the mate requires *k*-mer matches. These conditions help to control noise; however, they may exclude regions of interest. Each set of filtered trait reads is assembled *de novo* with SPAdes (v 3.11.1) [4] using the same *k*-mer size as the previous step (default 51). In some cases, a single erroneous *k*-mer, unique to the traits, can draw in reads on either side equating to approximately the insert size of the DNA in the sequencing library. Thus, the resulting assemblies are filtered to remove small contigs (default 300 bases) and low-coverage contigs (below 10×).

A scaffolding step is undertaken in an attempt to join together contigs that are located close by in the underlying genome, allowing gaps between contigs, equating to approximately the insert to be spanned. These spanned regions may contain *k*-mers that are not unique to the trait samples. To perform this scaffolding a *k*-mer database is generated for each trait assembly (FASTA format). No filtering is performed on the *k*-mer database.

For each trait sample, the raw reads in FASTQ format, plus their mates, which match any of these scaffolding *k*-mers, are extracted into new FASTQ files. These FASTQ files are then used to perform a second *de novo* assembly withSPAdes resulting in one assembly in FASTA format for each trait sample. A final filtering step of the assembled sequences is performed to remove low-coverage contigs, leaving one assembly in FASTA format for each trait sample. The most resource-intensive parts of the method, the *k*-mer analysis and the assembly, are parallelized using GNU parallel [10] to reduce the overall running time.

## Results

To evaluate the effectiveness of PlasmidTron, six experiments were performed comprising: (1) simulated reads to show the impact of copy number variation in identifying plasmids in *Salmonella enterica* serovar Weltevreden (*S*. Weltevreden), (2) identification of a novel AMR plasmid in *Salmonella enterica* serovar Typhi (*S.* Typhi)with subsequent validation using long-read sequencing, (3) investigating the effectiveness of different methods in recalling plasmid type sequences on real-world *S.* Weltevreden data (Supplementary Material), (4) investigating invasive versus carriage isolates in Klebsiella pneumoniae(Supplementary Material), (5) investigating liver abscess-associated ST23 *K. pneumoniae* compared with carriage isolates (Supplementary Material), and (6) an analysisof using PlasmidTron on a sample with multiple plasmids in *K. pneumoniae* (Supplementary Material). All experiments were performed using the Wellcome Trust Sanger Institute compute infrastructure, running Ubuntu 12.04.

## Impact of copy number variation

Simulated reads were generated to show the impact of copy number variation compared with other methods. A set of simulated perfect reads was generated. A reference genome, sequenced using the PacBio RSII for *S.* Weltevreden(accession number GCA_001409135), was used to generate perfect paired-end reads using FASTAQ (v3.15.0) (https://github.com/sanger-pathogens/fastaq) with a read length of 125 bases generated from a mean DNA fragment size of 400 bases. The reference is composed of a chromosome (5 062 936 bases) and a plasmid (98 756 bases), where the chromosome depth of coverage was fixed at 30×, and the plasmid depth of coverage was varied from 1 to 60× in steps of 2×. As a plasmid must be represented linearly in a FASTA file, the break pointfor the linearization was varied, in steps of 500 bases, to simulate a circular genome.

The results of PlasmidTron (v0.3.5) were compared with those of four other methods, Recycler (v0.6), Unicycler (v0.4.0),SPAdes(v3.10.0), and plasmidSPAdes (v3.10.0). SPAdes (v3.10.0) was used as the assembler for each of these methods. The Recycler method required read pre-processing steps usingbwa(v0.7.12) [11] and SAMtools (v0.1.19) [12]. SPAdes and Unicycler are not dedicated plasmid assembly programs, being agnostic to the underlying genomic structures; however, they provide a good baseline for what is possible to generate using non-specialist approaches. It is worth noting that the final plasmid sequences, if found, would be contained in a large collection of chromosome sequences. plasmidSPAdes and PlasmidTron are dedicated plasmid assemblers, and Recycler is a post-assembly plasmid analysis tool, with each employing a fundamentally different analysis strategy.

Each resulting assembly was measured based on the percentage of the known plasmid to be assembled, how fragmented the resulting plasmid assembly was, and the proportion of non-plasmid bases to plasmid bases within the assembled contigs (signal to noise ratio). The assemblies were blasted (v.2.6.0) [13] against the expected plasmid sequence, with an *E*-value of 0.0001. blast hits fewer than 200 bases long or with less than 90 % identity were excluded. Recycler identified no plasmids, which appears to be due to the large size of the plasmid genome and multiple repetitive insertion sequence elements in the underlying sequence, rendering the assembly graph too complex to unambiguously resolve.

**Fig. 2.**
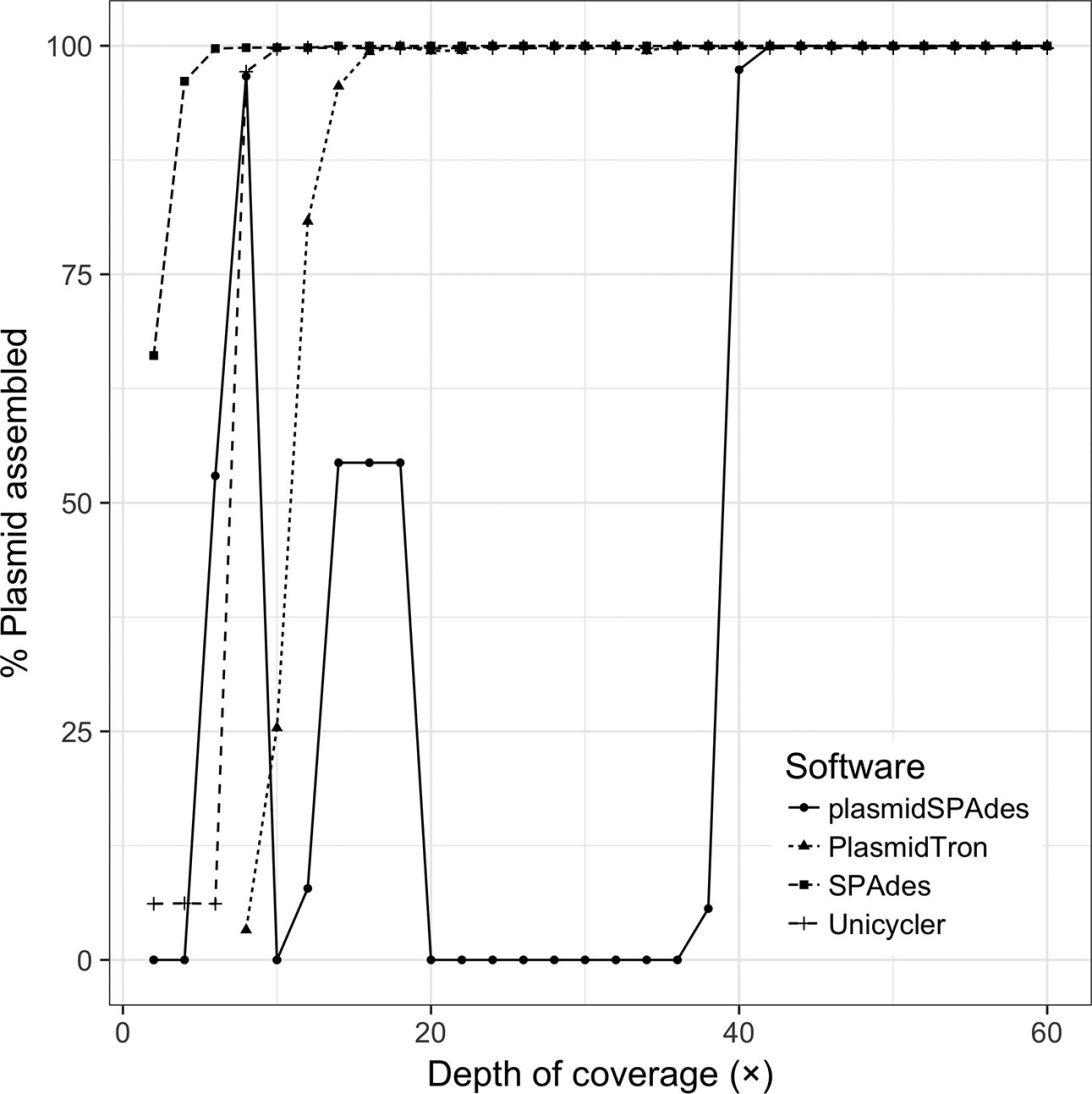
The percentage of the plasmid sequence that was assembled with different software applications as the depth of coverage of a plasmid increases in the raw data.

**Fig. 3.**
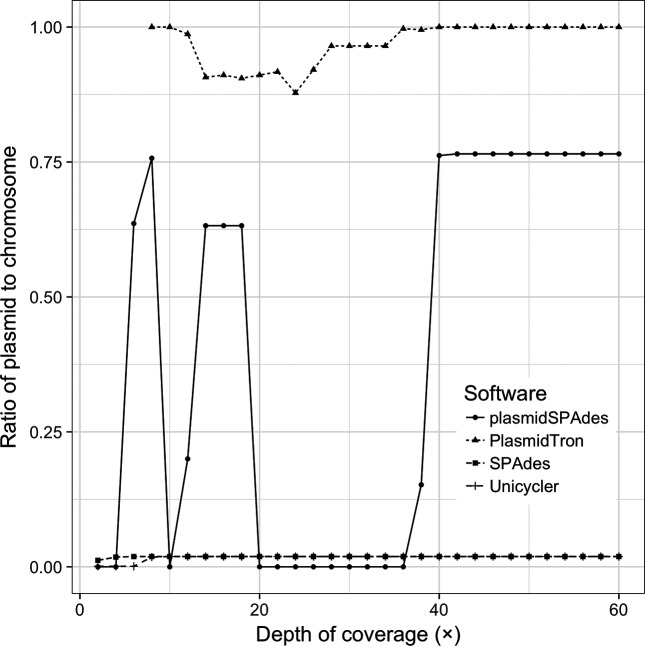
The ratio of the plasmid sequence to the chromosome sequence in the final assembly produced by each software application as the depth of coverage of the plasmid increases in the raw reads. This is akin to the signal to noise ratio.

Fig. 2 shows how, as the copy number of the plasmid in the input reads changes, the percentage of the plasmid recovered also changes. These data also show that plasmidSPAdes only identifies plasmid sequences above 40× depth of coverage, recovering the full plasmid sequence. plasmidSPAdes required a copy number difference of +/−0.5 times between the chromosome and the plasmid to identify the plasmid sequences. Above and below this level the plasmid copy number is too similar to the chromosome coverage such that the algorithm cannot distinguish between the two and the reads are filtered out. The SPAdes and Unicycler assemblers identify all of the plasmid sequence with less than 10× coverage; however, the plasmid sequences are fragmented and make up only ~1.9 % of the final assembled sequences, as shown in Fig. 3. PlasmidTron requires slightly more coverage (16×) to generate an assembly that covers the full plasmid sequence; however, there is no requirement for a difference in copy number between the chromosome and the plasmid, such as in plasmidSPAdes. At 16×, more than 90 % of the resulting contigs are plasmid-related sequences, increasing to 100 % at 40×. Taking the assemblies above 40×, where most assemblers reconstructed the plasmid, PlasmidTron consistently produces the most accurate assembly, with a mean of 7.9 single nucleotide polymorphisms, compared with plasmidSPAdes (10.9), Unicycler (167.4) and SPAdes (169.6). Full details are available in Table S1 (available with the online Supplementary Material).

## Outbreak AMR

PlasmidTron was used to analyse an outbreak of 87 *S.* Typhisamples with a resistance profile that had not been previously observed in haplotype H58 isolates (H58 isolates are representative of the dominant global multidrug-resistant clone of *S.* Typhi 4.3.1) [14]. Analysis using PlasmidFinder, as previously described, indicated that the antibiotic resistance may reside on an IncY plasmid, a plasmid type that has not been associated with this *S.* Typhi haplotype before. The chromosomes of six complete reference genomes for *S.* Typhi were used as the controls (accession numbers GCA_000195995, GCA_000007545, GCA_001157245, GCA_000245535, GCA_001302605, GCA_000385905) for PlasmidTron, and 87 Illumina sequenced outbreak samples were used as cases (TableS2). For each outbreak sample, PlasmidTron identified similar sequences, split over four or five contigs. One contig carried the IncY sequence and a second carried AMR genes. To verify this result we resequenced one isolate (ERS1670682) using long-read technology (MinION; Oxford Nanopore). This revealed that the four contigs identified here composed a single plasmid (accession number GCA_900185485.1), which was identical in all of the other outbreak strain genomes. Having these new references, we confirmed that the sequences generated by PlasmidTron recovered an average of 96 % of the plasmid sequence from short-read data. The fragmentation (mean 4.6) of the plasmid in the Illumina sequenced samples was due to repeats that could not be resolved with short-read sequencing. Overall, 65 % of the sequences in the resulting assemblies were part of the plasmid sequence, with the remainder resulting from a phage recombination in the main chromosome. This indicates the power of PlasmidTron to rapidly, accurately and cost-effectively extract sequences of clinical importance from short-read data alone.

## Conclusion

We can utilize the wealth of phenotypic data usually generated for bacterial population studies, be it routine diagnostics, surveillance or outbreak investigation, to reconstruct plasmids responsible for a particular phenotype. Rather than just identifying that an AMR or virulence gene exists in a sample, PlasmidTron can reconstruct both the gene and the genomic element harbouring the gene (e.g. a mobile element such as a plasmid), providing more insight into mechanisms associated with genes, such as the dispersal of the gene in the population. We demonstrated with simulated and real sequences that PlasmidTron reconstructs large plasmids more accurately than other methods. We present the results of a real outbreak of *S.* Typhi where PlasmidTron was used to identify the plasmid sequence carrying a novel AMR profile not previously described in *S.* Typhi H58/4.3.1, and validated the results using long-read sequencing. Additional experiments on *K. pneumoniae* demonstrated the effectiveness in the presence of multiple plasmids and complex phenotypes (outlined in detail in the Supplementary Material). Whilst plasmid assembly remains difficult with short reads, PlasmidTron allows phenotypic data to be utilized to greatly reduce the complexity of the challenge.

## Data bibliography

1. Parkhill J, Dougan G, James KD, Thomson NR, Pickard D *et al. Salmonella enterica* subsp. *enterica* serovar Typhi str. CT18, 2001, EMBL accession number GCA_000195995.
2. Deng W, Liou SR, Plunkett G III, Mayhew GF, Rose DJ *et al. Salmonella enterica* subsp. *enterica* serovar Typhi str. Ty2, 2006, EMBL accession number GCA_000007545.
3. Ong SY, Pratap CB, Wan X, Hou S, Abdul Rahman AY *et al. Salmonella enterica* subsp. *enterica* serovar Typhi str. P-stx-12, 2012, EMBL accession number GCA_000245535.
4. Xu D, Cisar JO, Poly F, Yang J, Albanese J *et al. Salmonella enterica* subsp. *enterica* serovar Typhi str. Ty21a, 2013, EMBL accession number GCA_000385905.
5. Muhamad Harish S, Sim KS, Najimudin N and Aziah I, *Salmonella enterica* subsp. *enterica* serovar Typhi str. PM016/13, 2015, EMBL accession number GCA_001302605.
6. Page AJ, *Salmonella enterica* serovar Weltevreden str. VNS10259, 2016, EMBL accession number GCA_001409135.
7. Page AJ, *Salmonella enterica* serovar Typhi str. 60006, 2017, EMBL accession number GCA_900185485.
8. Page AJ, *Salmonella enterica* serovar Typhi str. ERL12148, 2017, EMBL accession number GCA_001157245.

## Supplementary Data

282 Supplementary File 3

## Supplementary Data

77 Supplementary File 4
