# Supplementary material for: PlasmidTron: assembling the cause of phenotypes and genotypes from NGS data
Source: Microb Genom. 2018 Mar 12;4(3):e000164. doi: 10.1099/mgen.0.000164 (PMC5885016; doi:10.1099/mgen.0.000164)
Supplement: Supplementary File 3 [file mgen-4-164-s001.pdf]

Supplementary Material for "PlasmidTron: assembling the cause of phenotypes and genotypes from NGS data"

Recovery of typing sequences in S. Weltevreden

A real whole genome short read dataset of 114 samples of *S.* Weltevreden, was sequenced using Illumina HiSeq, as described in (Makendi et al. 2016). The samples represent a population of clonally related bacteria, with most sharing the same or a similar plasmid. The difference being the cargo genes that are carried on the plasmid, which varied greatly. To provide a point of comparison, and determine what plasmids were present in the input dataset, all of the samples were compared to the PlasmidFinder (Carattoli et al. 2014) database (retrieved 2017-07-25) using Ariba (v2.10.0) (Hunt et al. 2017), providing the Incompatibility (Inc) groups for each sample.

PlasmidFinder successfully identified one plasmid Inc group, $IncFII_S$, as present in 89.5% of all samples tested. *PlasmidSPAdes, Unicycler, SPAdes, Recycler* and *PlasmidTron* were provided with the same dataset and the resulting assembled contigs were searched for the $IncFII_S$ plasmid sequence, known to be present using blastn. Full details of these analysis are listed in Supplementary Table 6. Of the 106 samples known to have the $IncFII_S$ plasmid PlasmidFinder failed to identify the sequence in 4 instances, despite these plasmids being successfully assembled by 2 or more of the other algorithms. *SPAdes* and *Unicycler* assembled the $IncFII_S$ plasmid in 88.6% and 87.7% of samples. However, *PlasmidSPAdes* identified it in just 8.8% of sample data tested. Recycler failed to identify the plasmid sequence in any of the sequenced samples. *PlasmidTron* identified the plasmid sequence in 87.7% of cases where the chromosome sequence of *S.* Weltevreden strain VNS10259 was used as the control, giving identical results to *Unicycler*. The benefit though over *Unicycler* is that the plasmid contigs were highly enriched with majority of all of the assembled sequences corresponding to the $IncFII_S$ plasmid.

Klebsiella pneumoniae results

Klebsiella pneumoniae is a ubiquitous pathogen and a common cause of invasive infections in humans. *K. pneumoniae* is commonly found to contain multiple plasmids and it is these plasmids which have been associated with the transmission of antimicrobial resistance genes and virulence genes, posing a major threat to public health (Ejaz et al. 2017). The World Health Organization recently highlighted finding new treatments against MDR

Enterobacteriaceae (including *Klebsiella*) as priority 1 (critical) (http://www.who.int/mediacentre/news/releases/2017/bacteria-antibiotics-needed/en/).

We have reanalysed a dataset (Holt et al. 2015) on the genomic diversity, population structure, virulence, and antimicrobial resistance in *K. pneumoniae*, to evaluate the effectiveness of *PlasmidTron* where the underlying genomes are known to harbour multiple plasmids.

Invasive *K. pneumoniae* versus carriage

Two samples of *K. pneumoniae*, *1612* (accession number ERS012041) and *EW-85-R-MAN* (accession number ERS011857), with near identical core genes both from ST 36 and with capsule 149, display very different phenotypes (Holt et al. 2015). One sample (*1612*) caused invasive disease, the other (*EW-85-R-MAN*) did not cause disease (carriage). *PlasmidTron* was used to assemble sequences which were only found in the invasive sample to attempt to understand the underlying mechanisms of the pathogen. Comparing both samples with *PlasmidTron* using parameters '-k 31 -l 5000' gave an assembly of 37 contigs and 586,427 bases. The contigs were compared using *blastn* (Camacho et al. 2009) (version 2.7.0) to the non-redundant nucleotide (NT) GenBank database, with the top 20 largest contigs summarised in Supplementary Figure 1. Two previously identified *K. pneumoniae* plasmids, pSGH10 (accession number CP025081) and AR_0129_p2 (accession number CP021715.1), were present. As the plasmids both contained repetitive transposon sequences, full reconstruction of the underlying plasmid sequences was not possible using short reads alone. There are an additional 8 contigs which are associated with plasmids but their origin was ambiguous. This is most likely due to the recombined variants of a plasmid not being present in GenBank. Interestingly there are 3 complete novel sequences with no matches, even distant, in GenBank. Some chromosomal material is present in 7 contigs which can be depleted through the use of more control samples.

Size (bases)	pSGH10	AR_0129_p2	Other plasmid(s)	Novel	Chromosome
122370	■				
42551			■		
40201					■
39696	■				
37981					■
29478			■		
27545	■				
19562					■
18260					■

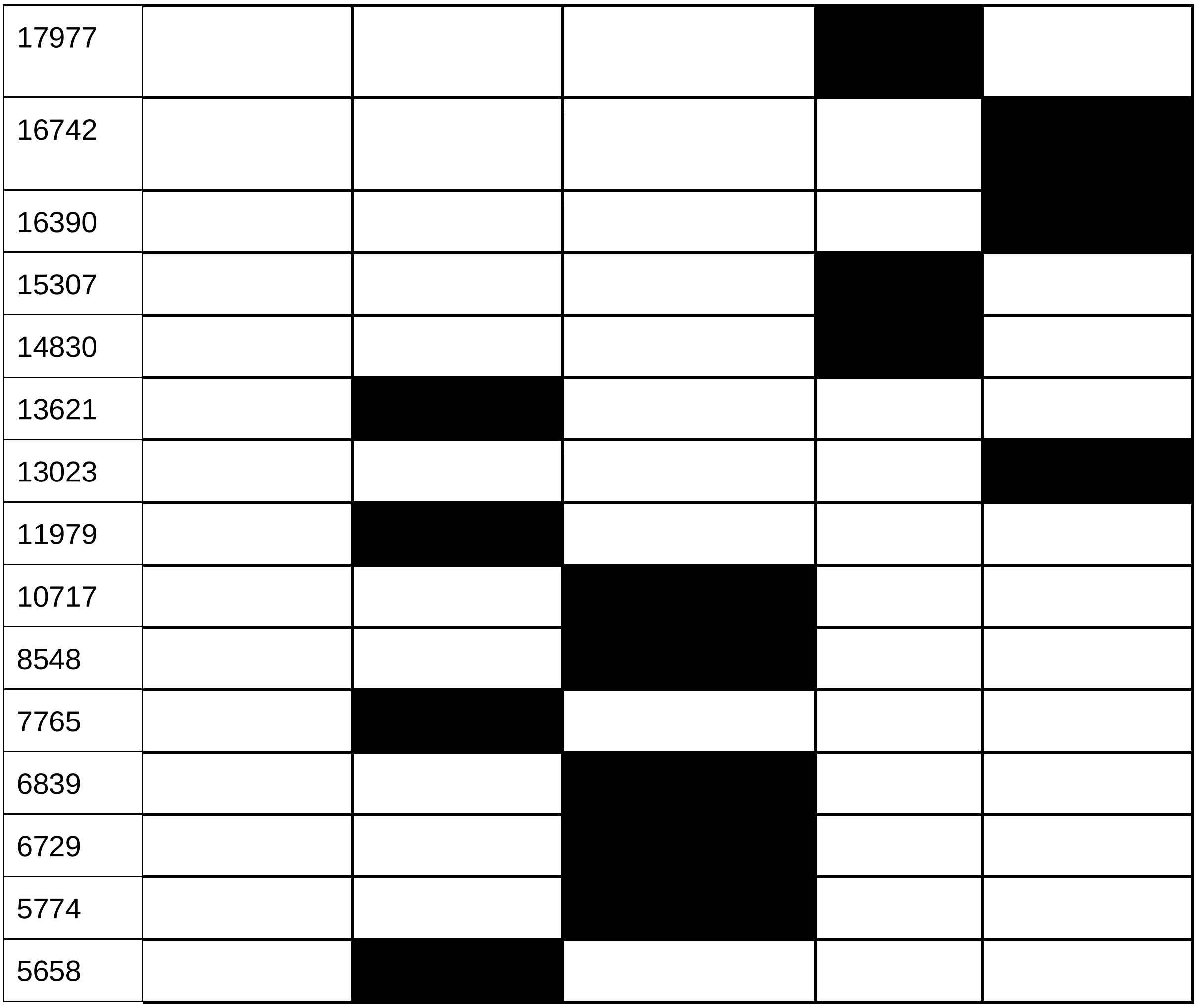

Supplementary Figure 1: *Classification of each contig based on results from blastn against the NT database, where the contigs are ordered by size as they are found in the output file of PlasmidTron. The black blocks indicate presence and white blocks absence. A contig was said to be present in a plasmid if the top hit was more than a 90% match in length and identity. A contig was said to be novel if no matches exceeding 30% identity and length were present.*

The *K. pneumoniae* plasmid pSGH10 (MC Lam et al. 2017) (MC Lam et al. 2017) in ST23 which a common cause of liver abscesses. The plasmid pSGH10 is mostly contained within 3 large contigs (189,611 bases) in the *PlasmidTron* assembly. Overall the *PlasmidTron* assembly covered 88.9% (205,993 out of 231,583 bases) of the pSGH10 plasmid and so can be said to be present in the sample. Supplementary Figure 2 shows an ACT (Carver et al. 2005) comparison of the *PlasmidTron* assembly against pSGH10, where the contigs are ordered by size.

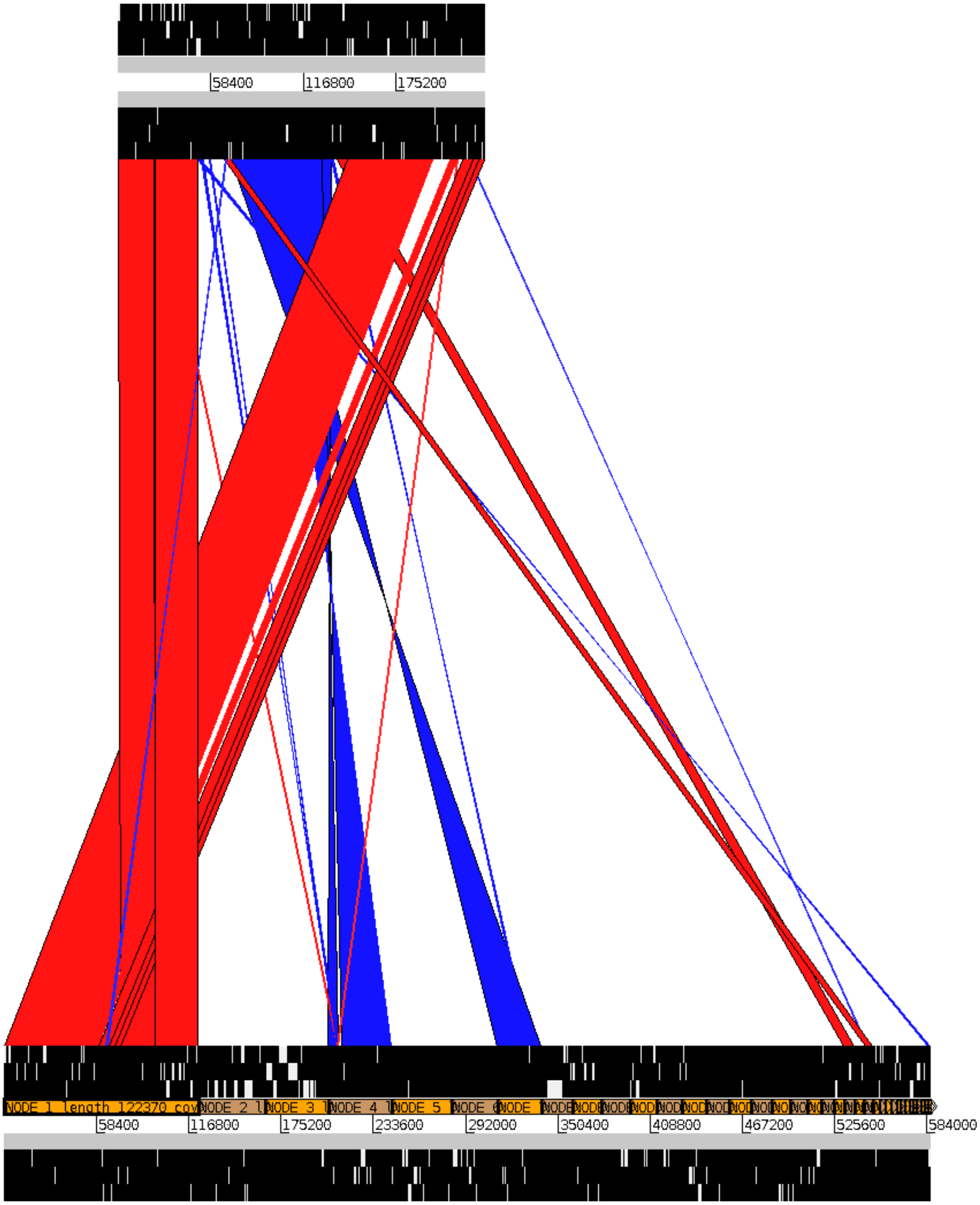

Supplementary Figure 2: *ACT comparison of the assembly produced by PlasmidTron (bottom) and the reference plasmid sequence* pSGH10 *(top). A red block indicates matching sequence, a blue block indicates a matching reversed sequence and no block indicates no matching sequence. The individual contigs of the PlasmidTron assembly are denoted by orange and brown blocks, ordered by contig size.*

The *K. pneumoniae* plasmid AR_0129_p2 carries extended-spectrum beta-lactamase (ESBL) resistance genes, with the resistance confirmed by phenotypic testing by the CDC (full antibiogram available from https://www.ncbi.nlm.nih.gov/biosample/SAMN04014970). AR_0129_p2 is contained within 4 contigs covering 80.2% (34,823 bases out of 43,378

bases) in the *PlasmidTron* assembly. Supplementary Figure 2 shows an ACT comparison of the *PlasmidTron* assembly against AR_0129_p2, where the contigs are ordered by size, zoomed in to the region with the major matches.

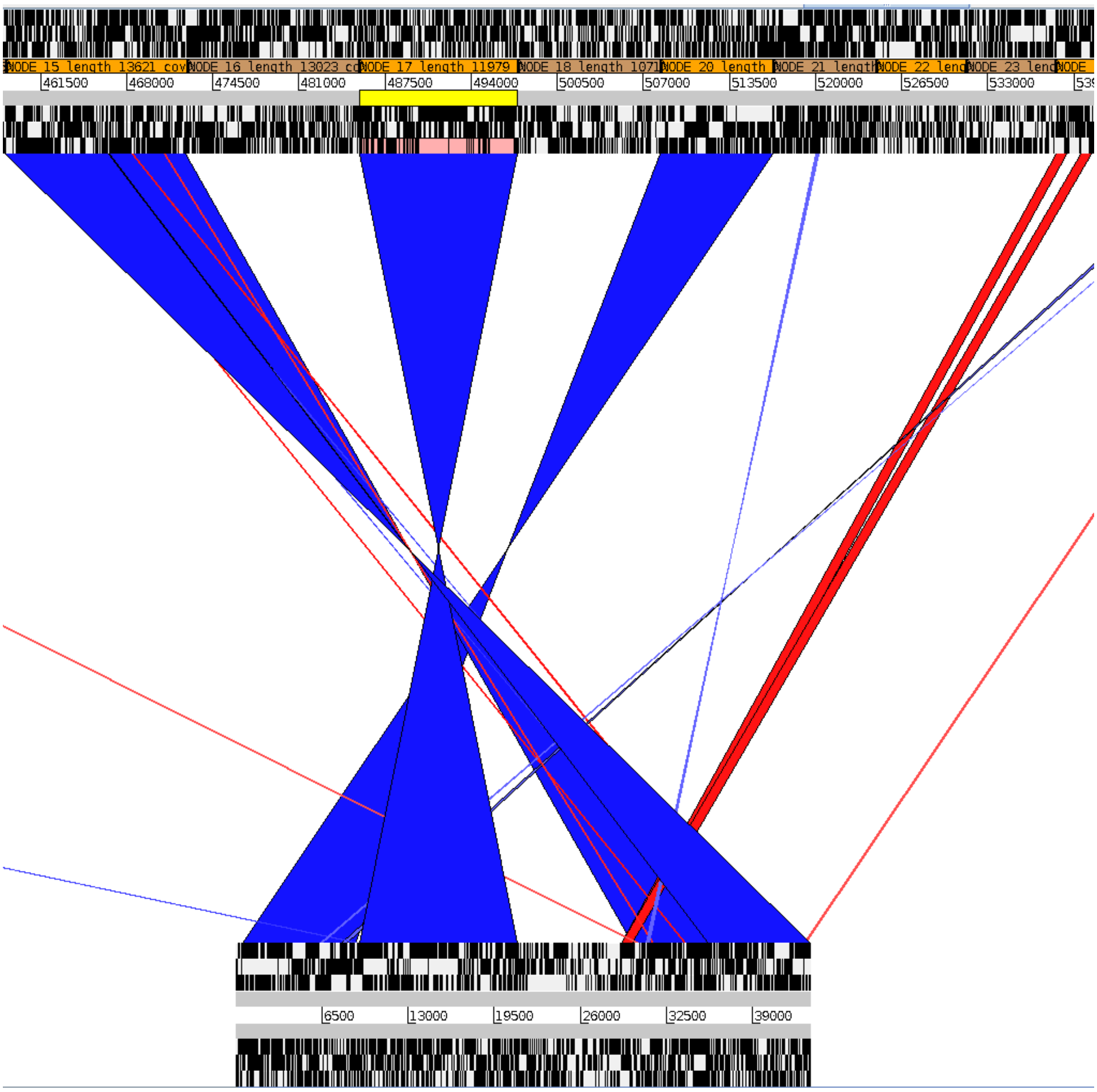

Supplementary Figure 3: *ACT comparison of the assembly produced by PlasmidTron (top) and the reference plasmid sequence AR_0129_p2 (bottom). A red block indicates matching sequence, a blue block indicates a matching reversed sequence and no block indicates no matching sequence. The individual contigs of the PlasmidTron assembly are denoted by orange and brown blocks, ordered by contig size. The PlasmidTron assembly has been zoomed in to focus just on the major blocks encompassing the AR_0129_p2 plasmid sequence.*

Given just 2 samples with an associated phenotype (invasive vs carriage) *PlasmidTron* has succeeded in assembling 2 fragmented plasmids, one of which is associated with hypervirulence and the other with antimicrobial resistance. These plasmids are strong candidates for having contributed to the more serious disease observed for the invasive sample.

What is different about liver abscess causing *K. pneumoniae* ST23 compared to carriage samples?

Sequence Type 23 (part of clonal group CG23) is associated with liver abscesses in humans. Six invasive disease samples from ST23 were compared to 17 carriage samples to see if *PlasmidTron* can determine any sequences which are universally present in the invasive samples and absent from the carriage samples. This data is drawn from (Holt et al. 2015). The invasive samples were collected between 2002 and 2008 from Asia. The carriage samples were collected between 2004 and 2007 and came from human (13) and bovine (4) samples, each one from a different ST. Extended metadata for the samples is available in Supplementary Table 5. The reference genome SGH10 (accession numbers CP025080, CP025081) (MC Lam et al. 2017) collected in 2015 was used for validation purposes as it is also ST23, confirmed using the *mlst* script (https://github.com/tseemann/mlst) with a database updated on 2017-07-09.

PlasmidTron was run with the parameters '--action intersection -k 61 -l 1000 -r 0.4' which is a strict mode, requiring a kmer to be present in all invasive samples with at least 10 times kmer coverage and absent from all carriage samples. The resulting assemblies had a mean of 649 kbases and a mean of 307 contigs. As a comparison a pan-genome was created of the same sets of samples. The raw reads were first assembled *de novo* using SPAdes (v3.11.1) (Bankevich et al. 2012) and annotated with PROKKA (version 1.10) (Seemann 2014). These annotated assemblies were then used as input to Roary with parameters '-e -n' (version 3.11.0) (Page et al. 2015). The cumulative running time for the assemblies, annotation and pan-genome generation was 146.45 hours. This compares to a running time for *PlasmidTron* of just 1.16 hours. The peak memory usage in both cases was under 1.5 GB. Roary identified 225 genes, equating to 224,221 coding bases, which were present in all invasive samples and absent from all carriage samples.

Comparing the Roary accessory genes to the assemblies produced by *PlasmidTron* using blastn (version 2.7.0) (Camacho et al. 2009) revealed that 4 invasive samples (66%) had blast matches to all 225 genes, the remaining samples had 224 and 223 genes with blast matches. When validated against the SGH10 reference genome and found a total of 83 (36.8%) of these genes were found to reside on plasmid pSGH10 and 142 (63.1%) on the chromosome. Supplementary Figure 4 shows the regions of pSGH10 which are unique to the invasive samples as identified by *PlasmidTron*. These regions (in blue) cover the iro (salmochelin), iuc (aerobactin) and rmpA/rmpA2 virulence genes (upregulators of capsule expression). A full discussion can be found in (MC Lam et al. 2017). *PlasmidTron* was able to identify these regions using phenotypic data alone, which is often the case when no complete reference genome is available. A limitation of the *PlasmidTron* method is that large segments of the pSGH10 plasmid are also present in 9 of the carriage strains, although they do not contain the virulence genes. This reduced the amount of the plasmid that could be assembled.

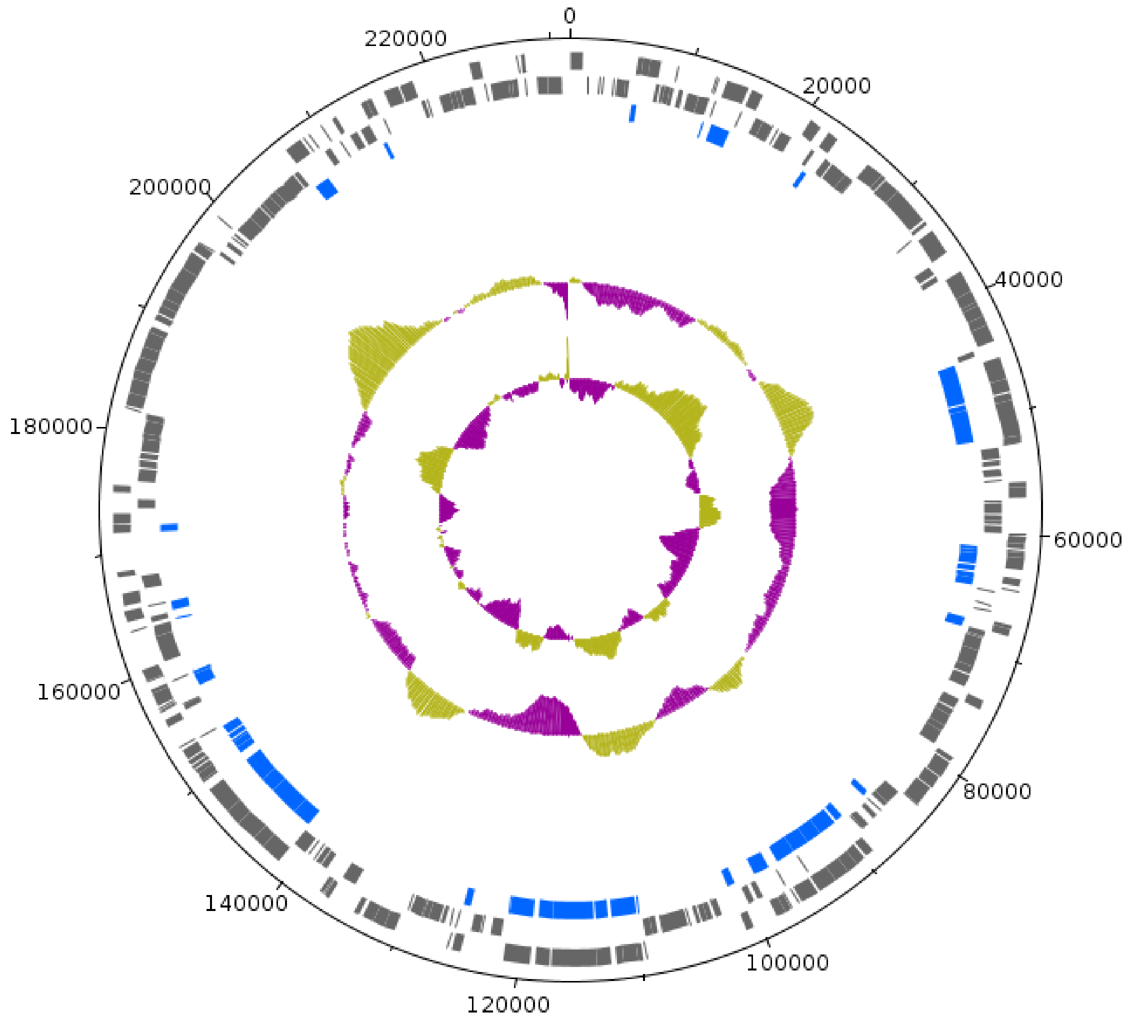

Supplementary Figure 4: *A circular plot of plasmid pSGH10 with the track (from inner to outer) consisting of GC skew (G-C/G+C), G+C content, genes identified by PlasmidTron as being unique to invasive samples (blue), coding regions on the reverse and forward strands (grey).*

Multiple plasmids in K. pneumoniae

K. pneumoniae samples can often have multiple plasmids. This makes it an ideal test case for *PlasmidTron*. The *K. pneumoniae* dataset from Holt et al. (Holt et al. 2015) was analysed using ARIBA (version 2.10.3) (Hunt et al. 2017) with the PlasmidFinder database (Carattoli et al. 2014) (accessed 05-12-2017) to identify all of the Incompatibility (Inc) groups in each sample. Sample AJ049 (accession number ERS005743) contained the most plasmid typing sequences (6) in the dataset so was chosen as a worst case scenario for *PlasmidTron*. All complete *K. pneumoniae* reference genomes (162) were downloaded from RefSeq (accessed 05-12-2017 and listed in Supplementary Table 4) and the chromosomes from each reference were extracted using *fasta_grep* (version 1) (https://github.com/sanger-

pathogens/fasta_grep) to use as controls. *PlasmidTron* was run with the parameter '-k 61 -d -l 2000' and the resulting assembly contained 520,314 bases with an N50 of 14,182, in 52 contigs. The assembly was compared to the PlasmidFinder database and all 6 plasmid typing sequences were recovered.

A blastn (version 2.7.0) (Camacho et al. 2009) with a minimum identity of 80% and a query coverage of 80% was performed on each of the 52 contigs against the nucleotide non-redundant (NT) database. Full details are listed in Supplementary Table 3. Of the 52 contigs, 41 (78.8%) were associated with plasmid sequences, all from *Enterobacteriaceae* encompassing *Klebsiella*, *Citrobacter*, *Enterobacter*, *Raoultella* and *Salmonella*. A further 2 (3.8%) were associated with chromosomal sequences from *Enterobacter xiangfangensis* and *Klebsiella michiganensis.* There was ambiguity which could not be resolved for 4 contigs (7.6%) which had a top hit in the *Raoultella ornithinolytica* (formerly *Klebsiella ornithinolytica*) chromosome (accession number CP017802.1), but every other blast hit matched to *K. pneumoniae* plasmids. This may be due to an assembly error with the deposited genome or an integration of a plasmid into the chromosome, however it is not possible to resolve this using short reads alone. Novel sequences, where there were no matches of any description in GenBank, accounted for 96Kb in 5 contigs (9.6%). Plasmid pKPN-332 (accession number CP014763.1) accounted for 6 contigs and pAUSMDU8141-1 (accession number CP022696.1) accounted for 14 contigs, so it is likely that both of these plasmids are present. The remaining 21 contigs were associated with a variety of plasmids but there was no clear pattern. As *PlasmidTron* does not use a database of reference plasmid sequences, it is not possible to unambiguously assign all contigs to particular plasmids. Whilst further post processing is required, *PlasmidTron* does rapidly identify the interesting parts of the accessory genome, substantially reducing the size of the data for the researcher.

References

Bankevich, Anton, Sergey Nurk, Dmitry Antipov, Alexey A. Gurevich, Mikhail Dvorkin, Alexander S. Kulikov, Valery M. Lesin, et al. 2012. "SPAdes: A New Genome Assembly Algorithm and Its Applications to Single-Cell Sequencing." *Journal of Computational Biology: A Journal of Computational Molecular Cell Biology* 19 (5):455–77.

Camacho, Christiam, George Coulouris, Vahram Avagyan, Ning Ma, Jason Papadopoulos, Kevin Bealer, and Thomas L. Madden. 2009. "BLAST+: Architecture and Applications." *BMC Bioinformatics* 10 (December):421.

Carattoli, Alessandra, Ea Zankari, Aurora García-Fernández, Mette Voldby Larsen, Ole Lund, Laura Villa, Frank Møller Aarestrup, and Henrik Hasman. 2014. "In Silico Detection and Typing of Plasmids Using PlasmidFinder and Plasmid Multilocus Sequence Typing." *Antimicrobial Agents and Chemotherapy* 58 (7):3895–3903.

Carver, Tim J., Kim M. Rutherford, Matthew Berriman, Marie-Adele Rajandream, Barclay G. Barrell, and Julian Parkhill. 2005. "ACT: The Artemis Comparison Tool." *Bioinformatics* 21 (16):3422–23.

Ejaz, Hasan, Nancy Wang, Jonathan J. Wilksch, Andrew J. Page, Hanwei Cao, Shruti Gujaran, Jacqueline A. Keane, et al. 2017. "Phylogenetic Analysis of Klebsiella Pneumoniae from Hospitalized Children, Pakistan." *Emerging Infectious Diseases* 23 (11):1872–75.

Holt, Kathryn E., Heiman Wertheim, Ruth N. Zadoks, Stephen Baker, Chris A. Whitehouse, David Dance, Adam Jenney, et al. 2015. "Genomic Analysis of Diversity, Population Structure, Virulence, and Antimicrobial Resistance in Klebsiella Pneumoniae, an Urgent

Threat to Public Health." *Proceedings of the National Academy of Sciences of the United States of America* 112 (27):E3574–81.
Hunt, Martin, Alison E. Mather, Leonor Sánchez-Busó, Andrew J. Page, Julian Parkhill, Jacqueline A. Keane, and Simon R. Harris. 2017. "ARIBA: Rapid Antimicrobial Resistance Genotyping Directly from Sequencing Reads." *Microbial Genomics* 3 (10):e000131.
Makendi, Carine, Andrew J. Page, Brendan W. Wren, Tu Le Thi Phuong, Simon Clare, Christine Hale, David Goulding, et al. 2016. "A Phylogenetic and Phenotypic Analysis of Salmonella Enterica Serovar Weltevreden, an Emerging Agent of Diarrheal Disease in Tropical Regions." *PLoS Neglected Tropical Diseases* 10 (2):e0004446.
MC Lam, Margaret, Kelly L. Wyres, Sebastian Duchene, Ryan R. Wick, Louise M. Judd, Yunn-Hwen Gan, Chu-Han Hoh, et al. 2017. "Population Genomics of Hypervirulent Klebsiella Pneumoniae Clonal Group 23 Reveals Early Emergence and Rapid Global Dissemination." *bioRxiv*. https://doi.org/10.1101/225359.
Page, Andrew J., Carla A. Cummins, Martin Hunt, Vanessa K. Wong, Sandra Reuter, Matthew T. G. Holden, Maria Fookes, Daniel Falush, Jacqueline A. Keane, and Julian Parkhill. 2015. "Roary: Rapid Large-Scale Prokaryote Pan Genome Analysis." *Bioinformatics* 31 (22):3691–93.
Seemann, Torsten. 2014. "Prokka: Rapid Prokaryotic Genome Annotation." *Bioinformatics* 30 (14):2068–69.
